# Di-*n*-butyl­ammonium 2-(3,5-di-*tert*-butyl-4-hydroxy­benzyl­sulfan­yl)nicotinate

**DOI:** 10.1107/S1600536808026202

**Published:** 2008-08-20

**Authors:** Shahirah Mansor, Wagee A. Yehye, Azhar Ariffin, Noorsaadah Abdul Rahman, Seik Weng Ng

**Affiliations:** a Department of Chemistry, University of Malaya, 50603 Kuala Lumpur, Malaysia

## Abstract

The asymmetric unit of the title compound, C_8_H_20_N^+^·C_21_H_26_NO_3_S^−^, contains two indpendent ion pairs which are disposed about a psuedo-inversion center, generating an ammonium–carboxylate N—H⋯O hydrogen-bonded four-component cluster. In the crystal structure, adjacent clusters are linked by hydr­oxy–carboxylate O—H⋯O hydrogen bonds, forming a chain.

## Related literature

For the applications of hindered phenol-based anti­oxidants, see: Kim & Lee (2003 ▶); Um *et al.* (2005 ▶).
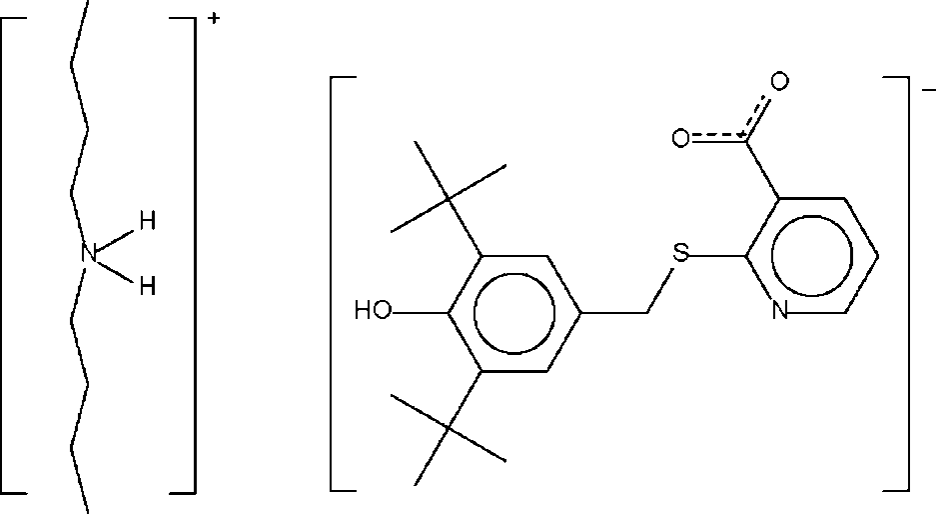


## Experimental

### 

#### Crystal data

C_8_H_20_N^+^·C_21_H_26_NO_3_S^−^
*M* *_r_* = 502.74
Orthorhombic, 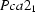

*a* = 23.4451 (3) Å
*b* = 18.7712 (3) Å
*c* = 12.9657 (2) Å
*V* = 5706.11 (15) Å^3^
*Z* = 8
Mo *K*α radiation
μ = 0.14 mm^−1^
*T* = 293 (2) K
0.25 × 0.15 × 0.05 mm

#### Data collection

Bruker SMART APEX diffractometer
Absorption correction: multi-scan (*SADABS*; Sheldrick, 1996 ▶) *T*_min_ = 0.965, *T*_max_ = 0.993
53030 measured reflections
13074 independent reflections
10355 reflections with *I* > 2σ(*I*)
*R*_int_ = 0.066

#### Refinement

*R*[*F* ^2^ > 2σ(*F* ^2^)] = 0.046
*wR*(*F* ^2^) = 0.110
*S* = 1.07
13074 reflections
647 parameters
1 restraint
H-atom parameters constrained
Δρ_max_ = 0.37 e Å^−3^
Δρ_min_ = −0.32 e Å^−3^
Absolute structure: Flack (1983 ▶), 6221 Friedel pairs
Flack parameter: 0.03 (6)

### 

Data collection: *APEX2* (Bruker, 2007 ▶); cell refinement: *SAINT* (Bruker, 2007 ▶); data reduction: *SAINT*; program(s) used to solve structure: *SHELXS97* (Sheldrick, 2008 ▶); program(s) used to refine structure: *SHELXL97* (Sheldrick, 2008 ▶); molecular graphics: *X-SEED* (Barbour, 2001 ▶); software used to prepare material for publication: *publCIF* (Westrip, 2008 ▶).

## Supplementary Material

Crystal structure: contains datablocks global, I. DOI: 10.1107/S1600536808026202/lh2682sup1.cif

Structure factors: contains datablocks I. DOI: 10.1107/S1600536808026202/lh2682Isup2.hkl

Additional supplementary materials:  crystallographic information; 3D view; checkCIF report

## Figures and Tables

**Table 1:** Hydrogen-bond geometry (Å, °)

*D*—H⋯*A*	*D*—H	H⋯*A*	*D*⋯*A*	*D*—H⋯*A*
N3—H32⋯O2	0.88	1.95	2.826 (2)	172
N3—H31⋯O5	0.88	1.99	2.820 (3)	157
N4—H42⋯O2	0.88	1.97	2.816 (3)	160
N4—H41⋯O5	0.88	1.96	2.834 (2)	172
O3—H3o⋯O1^i^	0.84	2.04	2.708 (3)	135
O6—H6o⋯O4^ii^	0.84	1.81	2.651 (3)	175

Symmetry codes: (i) 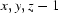; (ii) 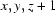.

**Fig. 1.**
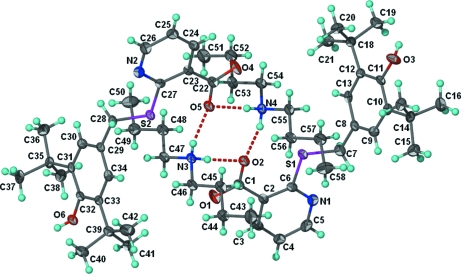
Thermal ellipsoid plot (Barbour, 2001) of O–H···O hydrogen-bonded dimeric structure of [(C4H9)2NH2] [C21H26NO3S] at the 70% probability level. Dashed lines denote the intra-ionic hydrogen bonds in the salt. Hydrogen atoms are drawn as spheres of arbitrary radius.

## supplementary crystallographic information

### Comment

The di-*n*-butylammonium salt of the substituted nicotinic acid (Scheme I, Fig. 1) is the precursor for the synthesis of hindered phenol-based antioxidants. Phenol-based antioxidants and their derivative have applications in industries such as pharmaceutical, textiles, plastics, polymers, oils, pesticides,dyestuffs, explosives, fluorescent-brightening industries. The salt was isolated along with the expected acid.

### Experimental

2-Mercaptonicotinic acid (1.50 g, 1 mmol), 2,6-di-*t*-butylphenol (2.00 g, 1 mmol) and paraformaldehyde (0.291 g,1 mmol) were intimately grounded and to the powder was added di-*n*-butylamine (0.09 ml). The slurry was heated to 373–383 K. The slurry solidified after an hour. The solid was purified by column chromatography, with chloroform as solvent, to give two products, one of which was 2-(3,5-di-*t*-butyl-4-hydroxybenzylthio)nicotinic acid and the other, di-*n*-butylammonium salt of the acid.

### Refinement

Carbon-bound H-atoms were placed in calculated positions (C—H 0.95 to 0.99 Å) and were included in the refinement in the riding model approximation, with *U*(H) set to 1.2–1.5*U*(C). The acid H-atom was located in a difference Fourier map, and was refined with a distance restraint of O–H 0.84±0.01 Å; its temperature factors were freely refined. The hydroxy H-atom was placed in a chemically sensible position, with a distance of more than 2 Å from the neighboring methyl H-atoms. The C–O–H fragment is then perpendicular to the aromatic ring.

### Crystal data

**Table:**

C_8_H_20_N^+^·C_21_H_26_NO_3_S^–^	*F*_000_ = 2192
*M**_r_* = 502.74	*D*_x_ = 1.170 Mg m^−^^3^
Orthorhombic, *P**c**a*2_1_	Mo *K*α radiation λ = 0.71073 Å
Hall symbol: P 2c -2ac	Cell parameters from 6621 reflections
*a* = 23.4451 (3) Å	θ = 2.2–25.1º
*b* = 18.7712 (3) Å	µ = 0.15 mm^−^^1^
*c* = 12.9657 (2) Å	*T* = 293 (2) K
*V* = 5706.11 (15) Å^3^	Prism, colorless
*Z* = 8	0.25 × 0.15 × 0.05 mm

### Data collection

**Table:**

Bruker SMART APEX diffractometer	13074 independent reflections
Radiation source: fine-focus sealed tube	10355 reflections with *I* > 2σ(*I*)
Monochromator: graphite	*R*_int_ = 0.066
*T* = 100(2) K	θ_max_ = 27.5º
ω scans	θ_min_ = 1.1º
Absorption correction: Multi-scan(SADABS; Sheldrick, 1996)	*h* = −30→30
*T*_min_ = 0.965, *T*_max_ = 0.993	*k* = −24→24
53030 measured reflections	*l* = −16→16

### Refinement

**Table:**

Refinement on *F*^2^	Hydrogen site location: inferred from neighbouring sites
Least-squares matrix: full	H-atom parameters constrained
*R*[*F*^2^ > 2σ(*F*^2^)] = 0.046	*w* = 1/[σ^2^(*F*_o_^2^) + (0.047*P*)^2^ + 0.106*P*] where *P* = (*F*_o_^2^ + 2*F*_c_^2^)/3
*wR*(*F*^2^) = 0.110	(Δ/σ)_max_ = 0.002
*S* = 1.07	Δρ_max_ = 0.37 e Å^−^^3^
13074 reflections	Δρ_min_ = −0.32 e Å^−^^3^
647 parameters	Extinction correction: none
1 restraint	Absolute structure: Flack (1983), 6221 Friedel pairs
Primary atom site location: structure-invariant direct methods	Flack parameter: 0.03 (6)
Secondary atom site location: difference Fourier map	

### Special details

**Table:**

Geometry. All e.s.d.'s (except the e.s.d. in the dihedral angle between two l.s. planes) are estimated using the full covariance matrix. The cell e.s.d.'s are taken into account individually in the estimation of e.s.d.'s in distances, angles and torsion angles; correlations between e.s.d.'s in cell parameters are only used when they are defined by crystal symmetry. An approximate (isotropic) treatment of cell e.s.d.'s is used for estimating e.s.d.'s involving l.s. planes.

### Fractional atomic coordinates and isotropic or equivalent isotropic displacement parameters (Å^2^)

**Table:**

	*x*	*y*	*z*	*U*_iso_*/*U*_eq_	
S1	0.61020 (3)	0.10851 (3)	0.49998 (5)	0.01813 (13)	
S2	0.37510 (2)	0.39163 (3)	0.74680 (5)	0.01700 (13)	
O1	0.55912 (8)	0.15648 (10)	0.82414 (14)	0.0307 (5)	
O2	0.54769 (7)	0.17288 (9)	0.65688 (14)	0.0215 (4)	
O3	0.59777 (7)	0.18381 (9)	0.01696 (14)	0.0275 (4)	
H3O	0.6043	0.1669	−0.0418	0.041*	
O4	0.41450 (9)	0.34101 (11)	0.41905 (14)	0.0367 (5)	
O5	0.43445 (7)	0.32878 (9)	0.58473 (13)	0.0206 (4)	
O6	0.41772 (7)	0.31263 (8)	1.21888 (13)	0.0190 (4)	
H6O	0.4154	0.3238	1.2815	0.028*	
N1	0.68358 (9)	0.02784 (11)	0.60001 (18)	0.0216 (5)	
N2	0.30600 (9)	0.48203 (10)	0.65446 (18)	0.0202 (5)	
N3	0.48194 (8)	0.27324 (10)	0.76764 (15)	0.0171 (4)	
H31	0.4621	0.2982	0.7228	0.021*	
H32	0.4994	0.2390	0.7338	0.021*	
N4	0.50031 (8)	0.22795 (10)	0.47395 (16)	0.0183 (5)	
H41	0.4817	0.2624	0.5053	0.022*	
H42	0.5205	0.2052	0.5207	0.022*	
C1	0.57288 (10)	0.14489 (12)	0.7329 (2)	0.0174 (5)	
C2	0.62184 (10)	0.09532 (12)	0.7132 (2)	0.0154 (5)	
C3	0.65056 (10)	0.06652 (12)	0.7969 (2)	0.0183 (5)	
H3	0.6393	0.0790	0.8649	0.022*	
C4	0.69525 (10)	0.02001 (13)	0.7826 (2)	0.0241 (6)	
H4	0.7155	0.0010	0.8398	0.029*	
C5	0.70982 (11)	0.00190 (14)	0.6839 (2)	0.0261 (6)	
H5	0.7402	−0.0309	0.6742	0.031*	
C6	0.64087 (10)	0.07440 (12)	0.6142 (2)	0.0168 (5)	
C7	0.64989 (12)	0.05976 (14)	0.4028 (2)	0.0224 (6)	
H7A	0.6913	0.0637	0.4163	0.027*	
H7B	0.6393	0.0087	0.4046	0.027*	
C8	0.63593 (10)	0.09092 (12)	0.2985 (2)	0.0171 (5)	
C9	0.67319 (10)	0.13750 (12)	0.2502 (2)	0.0195 (5)	
H9	0.7080	0.1492	0.2836	0.023*	
C10	0.66148 (10)	0.16767 (12)	0.1548 (2)	0.0174 (5)	
C11	0.60902 (10)	0.15045 (13)	0.1087 (2)	0.0167 (5)	
C12	0.56896 (10)	0.10404 (12)	0.1557 (2)	0.0149 (5)	
C13	0.58492 (10)	0.07448 (12)	0.2512 (2)	0.0178 (5)	
H13	0.5597	0.0421	0.2842	0.021*	
C14	0.70550 (10)	0.21645 (13)	0.1011 (2)	0.0189 (5)	
C15	0.75874 (11)	0.22665 (13)	0.1683 (2)	0.0273 (6)	
H15A	0.7861	0.2572	0.1322	0.041*	
H15B	0.7763	0.1802	0.1821	0.041*	
H15C	0.7479	0.2491	0.2337	0.041*	
C16	0.72503 (11)	0.18201 (13)	−0.0005 (2)	0.0269 (6)	
H16A	0.7571	0.2091	−0.0289	0.040*	
H16B	0.6934	0.1822	−0.0499	0.040*	
H16C	0.7370	0.1328	0.0127	0.040*	
C17	0.68062 (11)	0.29091 (12)	0.0801 (2)	0.0231 (6)	
H17A	0.7089	0.3199	0.0434	0.035*	
H17B	0.6710	0.3139	0.1457	0.035*	
H17C	0.6462	0.2864	0.0378	0.035*	
C18	0.50986 (10)	0.08924 (13)	0.1108 (2)	0.0195 (5)	
C19	0.51350 (11)	0.04930 (14)	0.0071 (2)	0.0269 (6)	
H19A	0.5330	0.0793	−0.0438	0.040*	
H19B	0.4749	0.0383	−0.0173	0.040*	
H19C	0.5349	0.0049	0.0165	0.040*	
C20	0.47803 (11)	0.16037 (14)	0.0993 (2)	0.0269 (6)	
H20A	0.5000	0.1923	0.0546	0.040*	
H20B	0.4733	0.1824	0.1673	0.040*	
H20C	0.4404	0.1518	0.0686	0.040*	
C21	0.47340 (11)	0.04226 (15)	0.1813 (2)	0.0304 (7)	
H21A	0.4913	−0.0047	0.1883	0.046*	
H21B	0.4353	0.0368	0.1513	0.046*	
H21C	0.4702	0.0646	0.2493	0.046*	
C22	0.40686 (10)	0.35717 (12)	0.5114 (2)	0.0181 (5)	
C23	0.36259 (10)	0.41265 (12)	0.53553 (19)	0.0143 (5)	
C24	0.33721 (10)	0.44858 (12)	0.4542 (2)	0.0184 (5)	
H24	0.3480	0.4374	0.3854	0.022*	
C25	0.29663 (10)	0.50023 (13)	0.4717 (2)	0.0208 (6)	
H25	0.2790	0.5250	0.4162	0.025*	
C26	0.28250 (11)	0.51463 (13)	0.5729 (2)	0.0225 (6)	
H26	0.2543	0.5499	0.5856	0.027*	
C27	0.34564 (10)	0.43232 (12)	0.63614 (19)	0.0141 (5)	
C28	0.33757 (11)	0.43887 (13)	0.8492 (2)	0.0170 (5)	
H28A	0.3463	0.4904	0.8461	0.020*	
H28B	0.2959	0.4325	0.8416	0.020*	
C29	0.35749 (10)	0.40803 (12)	0.95089 (19)	0.0148 (5)	
C30	0.32436 (10)	0.35929 (12)	1.0027 (2)	0.0173 (5)	
H30	0.2884	0.3464	0.9746	0.021*	
C31	0.34198 (10)	0.32795 (12)	1.0958 (2)	0.0155 (5)	
C32	0.39612 (10)	0.34766 (12)	1.13377 (19)	0.0157 (5)	
C33	0.43076 (10)	0.39769 (12)	1.08274 (19)	0.0146 (5)	
C34	0.40991 (10)	0.42713 (12)	0.9921 (2)	0.0162 (5)	
H34	0.4323	0.4616	0.9570	0.019*	
C35	0.30171 (10)	0.27660 (12)	1.1540 (2)	0.0183 (5)	
C36	0.24629 (11)	0.26426 (13)	1.0958 (2)	0.0243 (6)	
H36A	0.2546	0.2425	1.0287	0.036*	
H36B	0.2269	0.3099	1.0852	0.036*	
H36C	0.2217	0.2324	1.1358	0.036*	
C37	0.28590 (11)	0.31062 (13)	1.2593 (2)	0.0241 (6)	
H37A	0.2711	0.3588	1.2481	0.036*	
H37B	0.3199	0.3130	1.3031	0.036*	
H37C	0.2567	0.2816	1.2933	0.036*	
C38	0.32972 (11)	0.20349 (12)	1.1719 (2)	0.0216 (6)	
H38A	0.3375	0.1808	1.1053	0.032*	
H38B	0.3039	0.1733	1.2123	0.032*	
H38C	0.3656	0.2098	1.2096	0.032*	
C39	0.48994 (11)	0.41931 (13)	1.1241 (2)	0.0196 (5)	
C40	0.48624 (11)	0.44881 (13)	1.2354 (2)	0.0251 (6)	
H40A	0.4745	0.4106	1.2824	0.038*	
H40B	0.4582	0.4875	1.2379	0.038*	
H40C	0.5237	0.4670	1.2564	0.038*	
C41	0.53056 (11)	0.35538 (14)	1.1212 (2)	0.0261 (6)	
H41A	0.5331	0.3372	1.0505	0.039*	
H41B	0.5161	0.3178	1.1667	0.039*	
H41C	0.5685	0.3702	1.1446	0.039*	
C42	0.51695 (12)	0.47812 (15)	1.0582 (2)	0.0322 (7)	
H42A	0.5206	0.4615	0.9869	0.048*	
H42B	0.5548	0.4898	1.0856	0.048*	
H42C	0.4927	0.5206	1.0601	0.048*	
C43	0.65576 (12)	0.42326 (16)	0.7114 (2)	0.0344 (7)	
H43A	0.6851	0.4508	0.7476	0.052*	
H43B	0.6344	0.4547	0.6650	0.052*	
H43C	0.6739	0.3853	0.6713	0.052*	
C44	0.61514 (10)	0.39058 (14)	0.7898 (2)	0.0253 (6)	
H44A	0.6369	0.3587	0.8363	0.030*	
H44B	0.5982	0.4290	0.8322	0.030*	
C45	0.56770 (11)	0.34850 (13)	0.7389 (2)	0.0238 (6)	
H45A	0.5476	0.3795	0.6889	0.029*	
H45B	0.5844	0.3081	0.7002	0.029*	
C46	0.52529 (10)	0.32027 (13)	0.8164 (2)	0.0199 (5)	
H46A	0.5459	0.2932	0.8703	0.024*	
H46B	0.5058	0.3608	0.8502	0.024*	
C47	0.44247 (10)	0.24120 (13)	0.84408 (19)	0.0194 (5)	
H47A	0.4235	0.2798	0.8833	0.023*	
H47B	0.4648	0.2122	0.8935	0.023*	
C48	0.39737 (11)	0.19470 (13)	0.7948 (2)	0.0216 (6)	
H48A	0.4160	0.1552	0.7571	0.026*	
H48B	0.3753	0.2232	0.7444	0.026*	
C49	0.35703 (11)	0.16424 (14)	0.8757 (2)	0.0251 (6)	
H49A	0.3794	0.1343	0.9239	0.030*	
H49B	0.3407	0.2041	0.9159	0.030*	
C50	0.30874 (11)	0.12013 (15)	0.8326 (3)	0.0325 (7)	
H50A	0.2844	0.1035	0.8892	0.049*	
H50B	0.3244	0.0790	0.7955	0.049*	
H50C	0.2861	0.1492	0.7851	0.049*	
C51	0.33482 (13)	0.06566 (15)	0.5409 (2)	0.0332 (7)	
H51A	0.3061	0.0359	0.5068	0.050*	
H51B	0.3157	0.1030	0.5809	0.050*	
H51C	0.3580	0.0361	0.5871	0.050*	
C52	0.37294 (11)	0.09964 (14)	0.4602 (2)	0.0271 (6)	
H52A	0.3491	0.1271	0.4113	0.033*	
H52B	0.3926	0.0617	0.4208	0.033*	
C53	0.41734 (10)	0.14901 (13)	0.5081 (2)	0.0218 (6)	
H53A	0.3978	0.1894	0.5422	0.026*	
H53B	0.4391	0.1227	0.5613	0.026*	
C54	0.45836 (11)	0.17755 (13)	0.4273 (2)	0.0207 (6)	
H54A	0.4790	0.1373	0.3950	0.025*	
H54B	0.4366	0.2024	0.3726	0.025*	
C55	0.53960 (11)	0.25952 (13)	0.3961 (2)	0.0195 (5)	
H55A	0.5175	0.2897	0.3479	0.023*	
H55B	0.5577	0.2209	0.3557	0.023*	
C56	0.58557 (11)	0.30413 (13)	0.44713 (19)	0.0209 (6)	
H56A	0.6058	0.2751	0.4994	0.025*	
H56B	0.5678	0.3452	0.4827	0.025*	
C57	0.62798 (11)	0.33087 (15)	0.3670 (2)	0.0270 (6)	
H57A	0.6417	0.2899	0.3258	0.032*	
H57B	0.6081	0.3639	0.3195	0.032*	
C58	0.67879 (12)	0.36869 (14)	0.4135 (2)	0.0312 (7)	
H58A	0.7037	0.3860	0.3582	0.047*	
H58B	0.7000	0.3355	0.4574	0.047*	
H58C	0.6656	0.4091	0.4550	0.047*	

### Atomic displacement parameters (Å^2^)

**Table:**

	*U*^11^	*U*^22^	*U*^33^	*U*^12^	*U*^13^	*U*^23^
S1	0.0223 (3)	0.0195 (3)	0.0126 (3)	0.0055 (2)	−0.0010 (3)	−0.0002 (3)
S2	0.0206 (3)	0.0185 (3)	0.0120 (3)	0.0065 (2)	0.0000 (2)	0.0012 (3)
O1	0.0422 (12)	0.0361 (11)	0.0137 (10)	0.0189 (9)	0.0019 (9)	−0.0003 (8)
O2	0.0245 (10)	0.0234 (9)	0.0165 (9)	0.0082 (7)	−0.0021 (8)	−0.0004 (8)
O3	0.0347 (11)	0.0353 (10)	0.0124 (10)	−0.0099 (8)	−0.0040 (8)	0.0088 (8)
O4	0.0512 (14)	0.0457 (12)	0.0131 (10)	0.0302 (10)	0.0003 (9)	−0.0032 (9)
O5	0.0263 (10)	0.0229 (9)	0.0127 (9)	0.0090 (7)	−0.0012 (8)	0.0011 (7)
O6	0.0234 (9)	0.0213 (9)	0.0122 (9)	0.0025 (7)	−0.0018 (7)	0.0033 (7)
N1	0.0219 (11)	0.0222 (11)	0.0206 (12)	0.0052 (9)	0.0005 (9)	0.0030 (9)
N2	0.0218 (11)	0.0213 (10)	0.0177 (11)	0.0056 (8)	0.0006 (9)	0.0062 (9)
N3	0.0214 (11)	0.0170 (10)	0.0130 (11)	0.0064 (8)	−0.0013 (8)	−0.0009 (8)
N4	0.0196 (11)	0.0193 (10)	0.0159 (11)	0.0065 (8)	0.0018 (9)	−0.0007 (8)
C1	0.0208 (13)	0.0156 (12)	0.0156 (14)	0.0008 (9)	−0.0009 (10)	−0.0022 (10)
C2	0.0191 (12)	0.0130 (11)	0.0142 (13)	−0.0036 (9)	−0.0001 (10)	0.0004 (9)
C3	0.0208 (13)	0.0189 (12)	0.0152 (13)	−0.0036 (10)	−0.0007 (10)	−0.0003 (10)
C4	0.0211 (14)	0.0309 (15)	0.0204 (15)	0.0030 (11)	−0.0026 (11)	0.0073 (11)
C5	0.0209 (14)	0.0291 (14)	0.0283 (17)	0.0111 (11)	0.0008 (11)	0.0074 (12)
C6	0.0174 (12)	0.0154 (11)	0.0175 (14)	−0.0015 (9)	−0.0025 (10)	0.0014 (10)
C7	0.0264 (14)	0.0227 (14)	0.0179 (15)	0.0064 (11)	0.0001 (11)	0.0002 (11)
C8	0.0206 (14)	0.0169 (12)	0.0139 (14)	0.0063 (10)	0.0025 (10)	−0.0022 (10)
C9	0.0200 (12)	0.0201 (12)	0.0185 (13)	0.0050 (10)	−0.0025 (11)	−0.0048 (11)
C10	0.0175 (12)	0.0165 (12)	0.0182 (13)	0.0013 (9)	0.0035 (10)	−0.0005 (10)
C11	0.0216 (13)	0.0188 (12)	0.0098 (13)	0.0016 (10)	−0.0007 (10)	0.0001 (10)
C12	0.0150 (12)	0.0152 (12)	0.0144 (13)	−0.0004 (9)	0.0007 (10)	−0.0018 (10)
C13	0.0216 (13)	0.0150 (12)	0.0166 (14)	0.0013 (9)	0.0068 (11)	−0.0004 (11)
C14	0.0148 (12)	0.0220 (13)	0.0200 (14)	−0.0015 (9)	0.0036 (10)	0.0002 (11)
C15	0.0205 (14)	0.0315 (14)	0.0298 (16)	−0.0029 (11)	0.0008 (12)	−0.0017 (12)
C16	0.0268 (14)	0.0298 (14)	0.0241 (15)	−0.0038 (11)	0.0062 (12)	−0.0030 (12)
C17	0.0202 (13)	0.0234 (13)	0.0257 (15)	−0.0021 (10)	0.0045 (11)	0.0019 (11)
C18	0.0163 (12)	0.0237 (13)	0.0184 (13)	−0.0046 (10)	0.0012 (10)	−0.0027 (11)
C19	0.0294 (15)	0.0333 (15)	0.0180 (14)	−0.0087 (12)	−0.0018 (12)	−0.0056 (13)
C20	0.0198 (13)	0.0345 (15)	0.0264 (15)	0.0015 (11)	−0.0042 (12)	−0.0039 (12)
C21	0.0281 (15)	0.0381 (16)	0.0249 (16)	−0.0128 (12)	−0.0018 (12)	−0.0010 (12)
C22	0.0220 (13)	0.0175 (12)	0.0148 (13)	0.0010 (10)	0.0006 (11)	−0.0004 (10)
C23	0.0168 (13)	0.0124 (11)	0.0139 (13)	−0.0013 (10)	−0.0001 (10)	−0.0001 (9)
C24	0.0216 (13)	0.0203 (13)	0.0132 (13)	−0.0031 (10)	−0.0004 (10)	0.0023 (10)
C25	0.0211 (13)	0.0242 (13)	0.0171 (13)	0.0011 (10)	−0.0019 (10)	0.0079 (10)
C26	0.0258 (14)	0.0225 (13)	0.0192 (14)	0.0084 (10)	0.0002 (11)	0.0026 (10)
C27	0.0157 (12)	0.0128 (11)	0.0139 (13)	−0.0028 (9)	−0.0015 (10)	0.0034 (9)
C28	0.0193 (13)	0.0177 (12)	0.0139 (13)	0.0059 (10)	0.0027 (10)	−0.0019 (10)
C29	0.0176 (12)	0.0145 (12)	0.0124 (13)	0.0059 (9)	0.0003 (10)	−0.0026 (10)
C30	0.0174 (12)	0.0178 (12)	0.0167 (13)	0.0018 (9)	−0.0012 (10)	−0.0026 (11)
C31	0.0173 (12)	0.0153 (11)	0.0137 (13)	0.0028 (9)	0.0044 (10)	−0.0052 (10)
C32	0.0192 (12)	0.0175 (12)	0.0106 (13)	0.0058 (10)	0.0002 (10)	−0.0011 (10)
C33	0.0158 (12)	0.0146 (12)	0.0133 (13)	0.0004 (9)	0.0016 (9)	−0.0047 (10)
C34	0.0194 (12)	0.0137 (11)	0.0154 (14)	−0.0013 (9)	0.0034 (10)	−0.0028 (10)
C35	0.0171 (12)	0.0215 (12)	0.0164 (13)	−0.0004 (10)	0.0052 (10)	0.0003 (11)
C36	0.0195 (13)	0.0252 (13)	0.0281 (15)	−0.0024 (10)	0.0015 (12)	0.0024 (12)
C37	0.0230 (13)	0.0269 (13)	0.0224 (15)	−0.0029 (10)	0.0098 (11)	−0.0013 (11)
C38	0.0222 (13)	0.0187 (12)	0.0238 (15)	−0.0015 (10)	0.0028 (11)	0.0023 (11)
C39	0.0210 (13)	0.0217 (13)	0.0160 (14)	−0.0046 (10)	−0.0008 (10)	−0.0002 (10)
C40	0.0291 (14)	0.0243 (13)	0.0220 (16)	−0.0041 (11)	−0.0052 (12)	−0.0044 (12)
C41	0.0188 (13)	0.0399 (15)	0.0197 (14)	0.0019 (11)	−0.0016 (11)	−0.0036 (12)
C42	0.0297 (16)	0.0395 (17)	0.0275 (16)	−0.0155 (13)	−0.0038 (12)	0.0044 (13)
C43	0.0311 (16)	0.0406 (17)	0.0315 (17)	−0.0076 (13)	0.0051 (13)	−0.0065 (13)
C44	0.0255 (15)	0.0316 (15)	0.0187 (15)	0.0001 (11)	0.0000 (11)	0.0001 (12)
C45	0.0285 (14)	0.0231 (13)	0.0197 (15)	0.0007 (10)	0.0001 (12)	0.0003 (11)
C46	0.0249 (14)	0.0212 (13)	0.0135 (13)	0.0030 (10)	−0.0016 (11)	−0.0032 (10)
C47	0.0218 (13)	0.0247 (13)	0.0116 (13)	0.0021 (11)	0.0008 (10)	0.0016 (10)
C48	0.0249 (14)	0.0202 (13)	0.0197 (15)	0.0018 (11)	−0.0014 (11)	−0.0025 (11)
C49	0.0267 (15)	0.0246 (14)	0.0241 (16)	−0.0013 (11)	0.0000 (12)	−0.0015 (12)
C50	0.0300 (16)	0.0292 (15)	0.0385 (19)	−0.0023 (12)	−0.0103 (14)	0.0085 (13)
C51	0.0353 (17)	0.0401 (17)	0.0242 (16)	−0.0098 (13)	0.0034 (13)	−0.0026 (13)
C52	0.0275 (15)	0.0317 (15)	0.0223 (16)	−0.0045 (11)	−0.0012 (12)	−0.0022 (12)
C53	0.0280 (14)	0.0218 (13)	0.0156 (13)	−0.0009 (10)	0.0029 (11)	−0.0013 (11)
C54	0.0227 (14)	0.0233 (13)	0.0160 (13)	0.0022 (10)	−0.0025 (10)	−0.0009 (11)
C55	0.0225 (14)	0.0205 (13)	0.0156 (13)	0.0037 (10)	0.0022 (10)	0.0002 (10)
C56	0.0259 (14)	0.0225 (13)	0.0144 (14)	0.0022 (10)	−0.0009 (11)	0.0001 (10)
C57	0.0278 (15)	0.0309 (15)	0.0222 (15)	−0.0033 (12)	−0.0014 (12)	−0.0009 (12)
C58	0.0300 (16)	0.0272 (14)	0.0363 (18)	−0.0040 (12)	−0.0098 (13)	0.0063 (13)

### Geometric parameters (Å, °)

**Table:**

S1—C6	1.767 (3)	C29—C34	1.388 (3)
S1—C7	1.814 (3)	C30—C31	1.405 (4)
S2—C27	1.766 (2)	C30—H30	0.9500
S2—C28	1.823 (2)	C31—C32	1.411 (3)
O1—C1	1.245 (3)	C31—C35	1.546 (3)
O2—C1	1.264 (3)	C32—C33	1.407 (3)
O3—C11	1.370 (3)	C33—C34	1.387 (3)
O3—H3O	0.8400	C33—C39	1.542 (3)
O4—C22	1.248 (3)	C34—H34	0.9500
O5—C22	1.268 (3)	C35—C36	1.521 (4)
O6—C32	1.381 (3)	C35—C38	1.539 (3)
O6—H6O	0.8400	C35—C37	1.552 (4)
N1—C6	1.342 (3)	C36—H36A	0.9800
N1—C5	1.341 (3)	C36—H36B	0.9800
N2—C26	1.340 (3)	C36—H36C	0.9800
N2—C27	1.338 (3)	C37—H37A	0.9800
N3—C47	1.483 (3)	C37—H37B	0.9800
N3—C46	1.487 (3)	C37—H37C	0.9800
N3—H31	0.8800	C38—H38A	0.9800
N3—H32	0.8800	C38—H38B	0.9800
N4—C55	1.490 (3)	C38—H38C	0.9800
N4—C54	1.493 (3)	C39—C42	1.533 (4)
N4—H41	0.8800	C39—C41	1.533 (3)
N4—H42	0.8800	C39—C40	1.548 (4)
C1—C2	1.500 (3)	C40—H40A	0.9800
C2—C3	1.387 (3)	C40—H40B	0.9800
C2—C6	1.415 (4)	C40—H40C	0.9800
C3—C4	1.377 (3)	C41—H41A	0.9800
C3—H3	0.9500	C41—H41B	0.9800
C4—C5	1.367 (4)	C41—H41C	0.9800
C4—H4	0.9500	C42—H42A	0.9800
C5—H5	0.9500	C42—H42B	0.9800
C7—C8	1.509 (4)	C42—H42C	0.9800
C7—H7A	0.9900	C43—C44	1.521 (4)
C7—H7B	0.9900	C43—H43A	0.9800
C8—C13	1.379 (3)	C43—H43B	0.9800
C8—C9	1.385 (3)	C43—H43C	0.9800
C9—C10	1.388 (4)	C44—C45	1.515 (4)
C9—H9	0.9500	C44—H44A	0.9900
C10—C11	1.405 (3)	C44—H44B	0.9900
C10—C14	1.546 (3)	C45—C46	1.510 (4)
C11—C12	1.419 (3)	C45—H45A	0.9900
C12—C13	1.408 (4)	C45—H45B	0.9900
C12—C18	1.528 (3)	C46—H46A	0.9900
C13—H13	0.9500	C46—H46B	0.9900
C14—C15	1.535 (4)	C47—C48	1.513 (3)
C14—C16	1.536 (4)	C47—H47A	0.9900
C14—C17	1.539 (3)	C47—H47B	0.9900
C15—H15A	0.9800	C48—C49	1.524 (4)
C15—H15B	0.9800	C48—H48A	0.9900
C15—H15C	0.9800	C48—H48B	0.9900
C16—H16A	0.9800	C49—C50	1.510 (4)
C16—H16B	0.9800	C49—H49A	0.9900
C16—H16C	0.9800	C49—H49B	0.9900
C17—H17A	0.9800	C50—H50A	0.9800
C17—H17B	0.9800	C50—H50B	0.9800
C17—H17C	0.9800	C50—H50C	0.9800
C18—C21	1.531 (4)	C51—C52	1.517 (4)
C18—C20	1.537 (3)	C51—H51A	0.9800
C18—C19	1.541 (4)	C51—H51B	0.9800
C19—H19A	0.9800	C51—H51C	0.9800
C19—H19B	0.9800	C52—C53	1.525 (3)
C19—H19C	0.9800	C52—H52A	0.9900
C20—H20A	0.9800	C52—H52B	0.9900
C20—H20B	0.9800	C53—C54	1.519 (4)
C20—H20C	0.9800	C53—H53A	0.9900
C21—H21A	0.9800	C53—H53B	0.9900
C21—H21B	0.9800	C54—H54A	0.9900
C21—H21C	0.9800	C54—H54B	0.9900
C22—C23	1.503 (3)	C55—C56	1.517 (3)
C23—C24	1.386 (3)	C55—H55A	0.9900
C23—C27	1.413 (3)	C55—H55B	0.9900
C24—C25	1.377 (3)	C56—C57	1.523 (4)
C24—H24	0.9500	C56—H56A	0.9900
C25—C26	1.381 (4)	C56—H56B	0.9900
C25—H25	0.9500	C57—C58	1.512 (4)
C26—H26	0.9500	C57—H57A	0.9900
C28—C29	1.514 (4)	C57—H57B	0.9900
C28—H28A	0.9900	C58—H58A	0.9800
C28—H28B	0.9900	C58—H58B	0.9800
C29—C30	1.375 (3)	C58—H58C	0.9800
			
C6—S1—C7	101.00 (12)	C29—C34—C33	122.4 (2)
C27—S2—C28	101.10 (11)	C29—C34—H34	118.8
C11—O3—H3O	125.4	C33—C34—H34	118.8
C32—O6—H6O	129.0	C36—C35—C38	107.7 (2)
C6—N1—C5	117.9 (2)	C36—C35—C31	112.0 (2)
C26—N2—C27	117.7 (2)	C38—C35—C31	111.66 (19)
C47—N3—C46	112.50 (19)	C36—C35—C37	107.2 (2)
C47—N3—H31	109.1	C38—C35—C37	109.6 (2)
C46—N3—H31	109.1	C31—C35—C37	108.57 (19)
C47—N3—H32	109.1	C35—C36—H36A	109.5
C46—N3—H32	109.1	C35—C36—H36B	109.5
H31—N3—H32	107.8	H36A—C36—H36B	109.5
C55—N4—C54	112.64 (19)	C35—C36—H36C	109.5
C55—N4—H41	109.1	H36A—C36—H36C	109.5
C54—N4—H41	109.1	H36B—C36—H36C	109.5
C55—N4—H42	109.1	C35—C37—H37A	109.5
C54—N4—H42	109.1	C35—C37—H37B	109.5
H41—N4—H42	107.8	H37A—C37—H37B	109.5
O1—C1—O2	123.2 (2)	C35—C37—H37C	109.5
O1—C1—C2	117.9 (2)	H37A—C37—H37C	109.5
O2—C1—C2	118.9 (2)	H37B—C37—H37C	109.5
C3—C2—C6	116.7 (2)	C35—C38—H38A	109.5
C3—C2—C1	118.7 (2)	C35—C38—H38B	109.5
C6—C2—C1	124.6 (2)	H38A—C38—H38B	109.5
C4—C3—C2	120.7 (2)	C35—C38—H38C	109.5
C4—C3—H3	119.6	H38A—C38—H38C	109.5
C2—C3—H3	119.6	H38B—C38—H38C	109.5
C5—C4—C3	118.3 (2)	C42—C39—C41	107.1 (2)
C5—C4—H4	120.8	C42—C39—C33	111.6 (2)
C3—C4—H4	120.8	C41—C39—C33	110.2 (2)
N1—C5—C4	123.6 (2)	C42—C39—C40	106.6 (2)
N1—C5—H5	118.2	C41—C39—C40	109.7 (2)
C4—C5—H5	118.2	C33—C39—C40	111.6 (2)
N1—C6—C2	122.7 (2)	C39—C40—H40A	109.5
N1—C6—S1	115.14 (19)	C39—C40—H40B	109.5
C2—C6—S1	122.13 (18)	H40A—C40—H40B	109.5
C8—C7—S1	108.40 (17)	C39—C40—H40C	109.5
C8—C7—H7A	110.0	H40A—C40—H40C	109.5
S1—C7—H7A	110.0	H40B—C40—H40C	109.5
C8—C7—H7B	110.0	C39—C41—H41A	109.5
S1—C7—H7B	110.0	C39—C41—H41B	109.5
H7A—C7—H7B	108.4	H41A—C41—H41B	109.5
C13—C8—C9	119.1 (2)	C39—C41—H41C	109.5
C13—C8—C7	120.0 (2)	H41A—C41—H41C	109.5
C9—C8—C7	120.8 (2)	H41B—C41—H41C	109.5
C8—C9—C10	122.4 (2)	C39—C42—H42A	109.5
C8—C9—H9	118.8	C39—C42—H42B	109.5
C10—C9—H9	118.8	H42A—C42—H42B	109.5
C9—C10—C11	117.3 (2)	C39—C42—H42C	109.5
C9—C10—C14	120.8 (2)	H42A—C42—H42C	109.5
C11—C10—C14	121.9 (2)	H42B—C42—H42C	109.5
O3—C11—C10	115.6 (2)	C44—C43—H43A	109.5
O3—C11—C12	121.7 (2)	C44—C43—H43B	109.5
C10—C11—C12	122.6 (2)	H43A—C43—H43B	109.5
C13—C12—C11	116.3 (2)	C44—C43—H43C	109.5
C13—C12—C18	120.3 (2)	H43A—C43—H43C	109.5
C11—C12—C18	123.3 (2)	H43B—C43—H43C	109.5
C8—C13—C12	122.2 (2)	C45—C44—C43	112.3 (2)
C8—C13—H13	118.9	C45—C44—H44A	109.2
C12—C13—H13	118.9	C43—C44—H44A	109.2
C15—C14—C16	107.3 (2)	C45—C44—H44B	109.2
C15—C14—C17	107.2 (2)	C43—C44—H44B	109.2
C16—C14—C17	110.1 (2)	H44A—C44—H44B	107.9
C15—C14—C10	111.1 (2)	C46—C45—C44	112.1 (2)
C16—C14—C10	109.6 (2)	C46—C45—H45A	109.2
C17—C14—C10	111.39 (19)	C44—C45—H45A	109.2
C14—C15—H15A	109.5	C46—C45—H45B	109.2
C14—C15—H15B	109.5	C44—C45—H45B	109.2
H15A—C15—H15B	109.5	H45A—C45—H45B	107.9
C14—C15—H15C	109.5	N3—C46—C45	112.0 (2)
H15A—C15—H15C	109.5	N3—C46—H46A	109.2
H15B—C15—H15C	109.5	C45—C46—H46A	109.2
C14—C16—H16A	109.5	N3—C46—H46B	109.2
C14—C16—H16B	109.5	C45—C46—H46B	109.2
H16A—C16—H16B	109.5	H46A—C46—H46B	107.9
C14—C16—H16C	109.5	N3—C47—C48	112.8 (2)
H16A—C16—H16C	109.5	N3—C47—H47A	109.0
H16B—C16—H16C	109.5	C48—C47—H47A	109.0
C14—C17—H17A	109.5	N3—C47—H47B	109.0
C14—C17—H17B	109.5	C48—C47—H47B	109.0
H17A—C17—H17B	109.5	H47A—C47—H47B	107.8
C14—C17—H17C	109.5	C47—C48—C49	111.1 (2)
H17A—C17—H17C	109.5	C47—C48—H48A	109.4
H17B—C17—H17C	109.5	C49—C48—H48A	109.4
C12—C18—C21	112.5 (2)	C47—C48—H48B	109.4
C12—C18—C20	108.62 (19)	C49—C48—H48B	109.4
C21—C18—C20	106.7 (2)	H48A—C48—H48B	108.0
C12—C18—C19	111.7 (2)	C50—C49—C48	114.6 (2)
C21—C18—C19	105.8 (2)	C50—C49—H49A	108.6
C20—C18—C19	111.4 (2)	C48—C49—H49A	108.6
C18—C19—H19A	109.5	C50—C49—H49B	108.6
C18—C19—H19B	109.5	C48—C49—H49B	108.6
H19A—C19—H19B	109.5	H49A—C49—H49B	107.6
C18—C19—H19C	109.5	C49—C50—H50A	109.5
H19A—C19—H19C	109.5	C49—C50—H50B	109.5
H19B—C19—H19C	109.5	H50A—C50—H50B	109.5
C18—C20—H20A	109.5	C49—C50—H50C	109.5
C18—C20—H20B	109.5	H50A—C50—H50C	109.5
H20A—C20—H20B	109.5	H50B—C50—H50C	109.5
C18—C20—H20C	109.5	C52—C51—H51A	109.5
H20A—C20—H20C	109.5	C52—C51—H51B	109.5
H20B—C20—H20C	109.5	H51A—C51—H51B	109.5
C18—C21—H21A	109.5	C52—C51—H51C	109.5
C18—C21—H21B	109.5	H51A—C51—H51C	109.5
H21A—C21—H21B	109.5	H51B—C51—H51C	109.5
C18—C21—H21C	109.5	C51—C52—C53	112.2 (2)
H21A—C21—H21C	109.5	C51—C52—H52A	109.2
H21B—C21—H21C	109.5	C53—C52—H52A	109.2
O4—C22—O5	123.0 (2)	C51—C52—H52B	109.2
O4—C22—C23	117.9 (2)	C53—C52—H52B	109.2
O5—C22—C23	119.1 (2)	H52A—C52—H52B	107.9
C24—C23—C27	117.0 (2)	C54—C53—C52	111.5 (2)
C24—C23—C22	118.4 (2)	C54—C53—H53A	109.3
C27—C23—C22	124.6 (2)	C52—C53—H53A	109.3
C25—C24—C23	120.9 (2)	C54—C53—H53B	109.3
C25—C24—H24	119.5	C52—C53—H53B	109.3
C23—C24—H24	119.5	H53A—C53—H53B	108.0
C26—C25—C24	117.4 (2)	N4—C54—C53	111.2 (2)
C26—C25—H25	121.3	N4—C54—H54A	109.4
C24—C25—H25	121.3	C53—C54—H54A	109.4
N2—C26—C25	124.2 (2)	N4—C54—H54B	109.4
N2—C26—H26	117.9	C53—C54—H54B	109.4
C25—C26—H26	117.9	H54A—C54—H54B	108.0
N2—C27—C23	122.8 (2)	N4—C55—C56	111.3 (2)
N2—C27—S2	115.40 (19)	N4—C55—H55A	109.4
C23—C27—S2	121.81 (18)	C56—C55—H55A	109.4
C29—C28—S2	107.43 (16)	N4—C55—H55B	109.4
C29—C28—H28A	110.2	C56—C55—H55B	109.4
S2—C28—H28A	110.2	H55A—C55—H55B	108.0
C29—C28—H28B	110.2	C55—C56—C57	110.4 (2)
S2—C28—H28B	110.2	C55—C56—H56A	109.6
H28A—C28—H28B	108.5	C57—C56—H56A	109.6
C30—C29—C34	118.9 (2)	C55—C56—H56B	109.6
C30—C29—C28	120.4 (2)	C57—C56—H56B	109.6
C34—C29—C28	120.7 (2)	H56A—C56—H56B	108.1
C29—C30—C31	122.2 (2)	C58—C57—C56	113.4 (2)
C29—C30—H30	118.9	C58—C57—H57A	108.9
C31—C30—H30	118.9	C56—C57—H57A	108.9
C30—C31—C32	117.0 (2)	C58—C57—H57B	108.9
C30—C31—C35	120.0 (2)	C56—C57—H57B	108.9
C32—C31—C35	122.9 (2)	H57A—C57—H57B	107.7
O6—C32—C33	118.8 (2)	C57—C58—H58A	109.5
O6—C32—C31	118.9 (2)	C57—C58—H58B	109.5
C33—C32—C31	122.0 (2)	H58A—C58—H58B	109.5
C34—C33—C32	117.4 (2)	C57—C58—H58C	109.5
C34—C33—C39	120.4 (2)	H58A—C58—H58C	109.5
C32—C33—C39	122.1 (2)	H58B—C58—H58C	109.5
			
O1—C1—C2—C3	−2.0 (3)	C23—C24—C25—C26	0.1 (4)
O2—C1—C2—C3	177.9 (2)	C27—N2—C26—C25	−0.1 (4)
O1—C1—C2—C6	177.0 (2)	C24—C25—C26—N2	0.5 (4)
O2—C1—C2—C6	−3.1 (4)	C26—N2—C27—C23	−1.0 (4)
C6—C2—C3—C4	0.0 (3)	C26—N2—C27—S2	179.77 (18)
C1—C2—C3—C4	179.1 (2)	C24—C23—C27—N2	1.5 (3)
C2—C3—C4—C5	−1.3 (4)	C22—C23—C27—N2	−180.0 (2)
C6—N1—C5—C4	0.4 (4)	C24—C23—C27—S2	−179.28 (17)
C3—C4—C5—N1	1.1 (4)	C22—C23—C27—S2	−0.8 (3)
C5—N1—C6—C2	−1.7 (4)	C28—S2—C27—N2	−1.2 (2)
C5—N1—C6—S1	177.84 (19)	C28—S2—C27—C23	179.6 (2)
C3—C2—C6—N1	1.5 (4)	C27—S2—C28—C29	178.54 (17)
C1—C2—C6—N1	−177.5 (2)	S2—C28—C29—C30	−100.7 (2)
C3—C2—C6—S1	−177.98 (18)	S2—C28—C29—C34	77.8 (2)
C1—C2—C6—S1	3.0 (3)	C34—C29—C30—C31	−0.3 (4)
C7—S1—C6—N1	2.7 (2)	C28—C29—C30—C31	178.2 (2)
C7—S1—C6—C2	−177.7 (2)	C29—C30—C31—C32	−1.1 (3)
C6—S1—C7—C8	−170.89 (18)	C29—C30—C31—C35	176.7 (2)
S1—C7—C8—C13	−77.3 (3)	C30—C31—C32—O6	−173.2 (2)
S1—C7—C8—C9	101.1 (2)	C35—C31—C32—O6	9.1 (3)
C13—C8—C9—C10	−0.9 (4)	C30—C31—C32—C33	1.5 (3)
C7—C8—C9—C10	−179.4 (2)	C35—C31—C32—C33	−176.2 (2)
C8—C9—C10—C11	1.4 (4)	O6—C32—C33—C34	174.2 (2)
C8—C9—C10—C14	−176.9 (2)	C31—C32—C33—C34	−0.5 (3)
C9—C10—C11—O3	177.0 (2)	O6—C32—C33—C39	−5.3 (3)
C14—C10—C11—O3	−4.6 (3)	C31—C32—C33—C39	180.0 (2)
C9—C10—C11—C12	−0.3 (4)	C30—C29—C34—C33	1.4 (4)
C14—C10—C11—C12	178.0 (2)	C28—C29—C34—C33	−177.1 (2)
O3—C11—C12—C13	−178.4 (2)	C32—C33—C34—C29	−1.0 (3)
C10—C11—C12—C13	−1.2 (3)	C39—C33—C34—C29	178.5 (2)
O3—C11—C12—C18	−2.1 (4)	C30—C31—C35—C36	2.6 (3)
C10—C11—C12—C18	175.1 (2)	C32—C31—C35—C36	−179.8 (2)
C9—C8—C13—C12	−0.7 (4)	C30—C31—C35—C38	123.5 (2)
C7—C8—C13—C12	177.7 (2)	C32—C31—C35—C38	−58.9 (3)
C11—C12—C13—C8	1.7 (3)	C30—C31—C35—C37	−115.6 (2)
C18—C12—C13—C8	−174.7 (2)	C32—C31—C35—C37	62.1 (3)
C9—C10—C14—C15	−1.9 (3)	C34—C33—C39—C42	4.3 (3)
C11—C10—C14—C15	179.8 (2)	C32—C33—C39—C42	−176.2 (2)
C9—C10—C14—C16	116.5 (3)	C34—C33—C39—C41	−114.5 (2)
C11—C10—C14—C16	−61.8 (3)	C32—C33—C39—C41	65.0 (3)
C9—C10—C14—C17	−121.4 (3)	C34—C33—C39—C40	123.4 (2)
C11—C10—C14—C17	60.4 (3)	C32—C33—C39—C40	−57.1 (3)
C13—C12—C18—C21	1.0 (3)	C43—C44—C45—C46	−176.2 (2)
C11—C12—C18—C21	−175.1 (2)	C47—N3—C46—C45	176.0 (2)
C13—C12—C18—C20	118.9 (2)	C44—C45—C46—N3	−174.5 (2)
C11—C12—C18—C20	−57.3 (3)	C46—N3—C47—C48	178.62 (19)
C13—C12—C18—C19	−117.8 (3)	N3—C47—C48—C49	−178.7 (2)
C11—C12—C18—C19	66.0 (3)	C47—C48—C49—C50	177.0 (2)
O4—C22—C23—C24	−7.1 (4)	C51—C52—C53—C54	175.1 (2)
O5—C22—C23—C24	173.2 (2)	C55—N4—C54—C53	−177.43 (19)
O4—C22—C23—C27	174.4 (2)	C52—C53—C54—N4	177.9 (2)
O5—C22—C23—C27	−5.3 (4)	C54—N4—C55—C56	−173.89 (19)
C27—C23—C24—C25	−1.0 (3)	N4—C55—C56—C57	175.4 (2)
C22—C23—C24—C25	−179.6 (2)	C55—C56—C57—C58	−173.4 (2)

### Hydrogen-bond geometry (Å, °)

**Table:**

*D*—H···*A*	*D*—H	H···*A*	*D*···*A*	*D*—H···*A*
N3—H32···O2	0.88	1.95	2.826 (2)	172
N3—H31···O5	0.88	1.99	2.820 (3)	157
N4—H42···O2	0.88	1.97	2.816 (3)	160
N4—H41···O5	0.88	1.96	2.834 (2)	172
O3—H3o···O1^i^	0.84	2.04	2.708 (3)	135
O6—H6o···O4^ii^	0.84	1.81	2.651 (3)	175

Symmetry codes: (i) *x*, *y*, *z*−1; (ii) *x*, *y*, *z*+1.

## References

[bb1] Barbour, L. J. (2001). *J. Supramol. Chem.* **1**, 189–191.

[bb2] Bruker (2007). *APEX2* and *SAINT* Bruker AXS Inc., Madison, Wisconsin, USA.

[bb3] Flack, H. D. (1983). *Acta Cryst.* A**39**, 876–881.

[bb4] Kim, T. H. & Lee, N. (2003). *Bull. Kor. Chem. Soc.*, **24**, 1809–1813.

[bb5] Sheldrick, G. M. (1996). *SADABS* University of Göttingen, Germany.

[bb6] Sheldrick, G. M. (2008). *Acta Cryst.* A**64**, 112–122. 10.1107/S0108767307043930 18156677

[bb7] Um, S.-I., Kang, Y.-H. & Lee, J.-Y. (2005). *Dyes Pigm* **64**, 93–99.

[bb8] Westrip, S. P. (2008). *publCIF* In preparation.

